# Towards brain-tissue-like biomaterials

**DOI:** 10.1038/s41467-020-17245-x

**Published:** 2020-07-09

**Authors:** Eneko Axpe, Gorka Orive, Kristian Franze, Eric A. Appel

**Affiliations:** 1 Department of Materials Science & Engineering, Stanford University, Stanford, CA 94305 USA; 2 Space Biosciences Division, Ames Research Center, NASA, Mountain View, CA 94035 USA; 3 NanoBioCel Group, Laboratory of Pharmaceutics, School of Pharmacy, University of the Basque Country UPV/EHU, Vitoria-Gasteiz, Spain; 4 Biomedical Research Networking Centre in Bioengineering, Biomaterials and Nanomedicine (CIBER-BBN), Vitoria-Gasteiz, Spain; 5 University Institute for Regenerative Medicine and Oral Implantology-UIRMI (UPV/EHU-Fundación Eduardo Anitua), Vitoria, Spain; 6 Discovery Tower, Singapore Eye Research Institute, The Academia, Singapore, Singapore; 7 Department of Physiology, Development and Neuroscience, University of Cambridge, Cambridge, CB2 3DY UK; 8 Department of Bioengineering, Stanford University, Stanford, CA 94305 USA

**Keywords:** Neuroscience, Biomedical engineering, Chemical engineering, Biomaterials

## Abstract

Many biomaterials have been developed which aim to match the elastic modulus of the brain for improved interfacing. However, other properties such as ultimate toughness, tensile strength, poroviscoelastic responses, energy dissipation, conductivity, and mass diffusivity also need to be considered.

## Need for brain-tissue-like biomaterials

Creating biomaterials resembling brain tissue is required for many emerging technologies. Neural probes in brain–machine interfaces^1,2^, microphysiological models of neurological diseases^3^, scaffolds for neural tissue engineering^4,5^, brain organoids^6^, and brain proxies^7^ (e.g., for studying traumatic brain injury while reducing the need for animal testing) need to mimic the physical properties of brain tissue to be successfully applied^8^. For in vivo applications, a mechanical match between implants and the surrounding brain tissue can minimize immune response and implant rejection due to the foreign-body response^9,10^. Recapitulation of the native environment of neurons and glial cells in vitro is crucial for their appropriate differentiation, motility, function, and proliferation either to expand cells for therapeutic applications or to study cellular responses to chemical signals and new treatments in vitro^11,12^.

## Physical properties of the brain

The brain is a complex tissue that is anisotropic and remarkably soft; indeed, it is one of the softest organs in the body. And when things are soft, they get hard to engineer. Materials scientists have found it challenging to fabricate functional biomaterials resembling the low stiffness of brain tissue. One important question that remains: why is brain tissue so soft?

The brain’s unique architecture causes it to respond mechanically as a poroviscoelastic material, whereby the cerebrospinal fluid can be excreted from the brain’s matrix under compression^13^. This response contributes to the apparent bulk softness of the brain, regardless of the stiffness of the elements arranged throughout the tissue^14–16^. In microscopic measurements, brain is also exceptionally soft; brain parenchyma contains very little fibrous collagen I, which correlates strongly with the stiffness of different organs^17^. Furthermore, it contains large amounts of different proteogylcans^18,19^, heavily glycosylated proteins that bind water. This makes the water content in brain relatively high, between 73 and 85% of the total mass.

On the other hand, myelin acts as an insulator material, which is composed mainly of lipids. In fact, lipids account for roughly 60% of the total dry weight of the brain. As the myelin content of neural tissue scales with the tissue’s stiffness^20^, differential myelination contributes to the mechanical heterogeneity of brain and spinal cord tissue.

## Limitations of current research

Characterizing the stiffness of the brain with traditional tools is often unreliable, because of the low bending stiffness of brain tissue. Recently, different experimental techniques such as atomic force microscopy^21^, microindentation^22,23^, rheology^24^, and magnetic resonance elastography^25^ have been used in vivo and ex vivo, under dry and wet conditions, and with different boundary and loading conditions. Using these various techniques the elastic modulus of brain tissue is typically shown to be in the range of few hundreds of Pa to kPa, yet the experimental methods, animal models, and conditions used to conduct these tests diverge substantially from lab to lab, inducing a high degree of variability in measurement outcomes. Consequently, comparison between various studies can be highly unreliable. Moreover, the mechanical properties of the different parts of the brain have not been thoroughly characterized to date. It is still unclear, for example, whether gray matter or white matter is stiffer as different studies show contradictory results. In-depth studies characterizing the stiffness of brain tissue at all scales, from bulk to the nanoscale (scale at which neurons and glial cells sense)^26–28^, and under physiological conditions are therefore essential for the field to move forward. Further, it is crucial that the field coalesce around standardized methods for characterization at these various scales to ensure reliable comparisons can be made between studies. We encourage different laboratories to join forces to generate standardized experimental methodologies and perhaps perform round robin testing to produce reliable interlaboratory comparisons.

## Mechanical mismatch

**Fig. 1:**
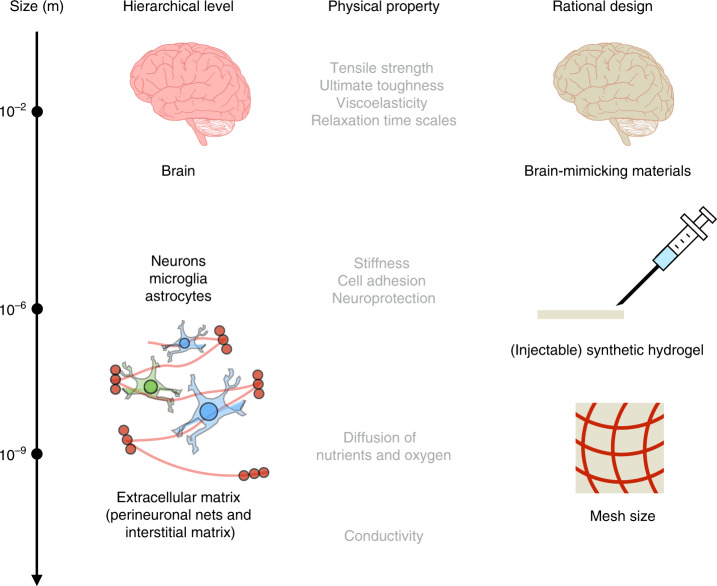
Physical features of brain tissue that need to be resembled at different scales. The extracellular matrix of the brain (perinueonal net and the neural interstitial matrix) and its protein nanoscale building blocks give brain tissue its physical properties that differ from region to region. Resembling at different scales mechanical properties such as the tensile strength, ultimate toughness, viscoelasticity, relaxation times, stiffness, cell adhesion, and well as other physical features such diffusion of nutrients and oxygen and conductivity are key for the rational design of brain tissue mimicking hydrogels. Biomaterials comprehensively capturing the diverse properties of brain tissue can propel applications such as neural probes for brain–machine interfaces, brain organoids for studying neurogenesis or drug screening and injectable materials to substitute current hard materials to treat certain types of brain tumors such as glioblastoma multiforme. Brain graphic adapted from the following, under a CC BY 3.0 licence: https://webstockreview.net/image/clipartbraintransparentbackground/426010.html.

When researchers discuss the mechanical mismatch between engineered biomaterials and brain tissue, they often compare stiffness alone. *E pluribus unum*, reducing the complexity of the brain to this one mechanical parameter, while simple, is nevertheless taking a very limited view. The field must move towards determining other mechanical properties crucial for the successful use of biomaterials in these applications. Apart from stiffness, the tensile strength, ultimate toughness, viscoelasticity, relaxation time-scales, adhesion, and structural parameters contributing to the diffusion of solutes are essential properties that must be characterized in greater depth  (Fig. 1). It is known that mechanical mismatches can cause primary injuries that start at the cellular scale, but the degree to which a mechanical mismatch can cause mechano-chemical injuries through the activation of apoptotic and/or necrotic cellular pathways remains to be understood. Materials developed in the future will need to offer extremely high neuroprotection, as it has been shown that injuries can initiate at small local deformations (shear strains of just 14% in impacts at critical and supercritical strain rates)^29^.

Only once we deeply understand the properties of brain tissue, we will be able to develop biomaterials resembling its complex properties. In this sense, diffusion of solutes through biomaterials will be critical. For instance, previous studies have correlated the mechanical properties of hydrogel scaffolds to the differentiation of stem cells into neurons^30^. Yet, the mesh size is often overlooked, even if it is known to be a main contributor to the diffusion properties of solutes in hydrogels^31^. It will undoubtedly be essential for biomaterials to imitate the transport of oxygen, nutrients, and therapeutics in real brain tissue. One simple characterization technique we would advocate for adoption in assessing diffusivity is fluorescence recovery after photobleaching.

Biodegradation times should be also considered for the design of brain-tissue-like neural probes or biomaterials for neural tissue engineering. Unfortunately, degradation studies of implants in the brain under physiological conditions are very scarce. Fluids in the brain contain proteins, peptides, sugars, and ions that interact, and degrade, the material inserted. Furthermore, inflamed brain tissue can produce reactive oxygen species^32^ that can contribute to implant degradation. In addition to mechanical testing, we need to design new methods to resemble biodegradation under natural physiological conditions within the brain (Fig. 1).

## A more exhaustive, multi-scale design

So far, there are no materials that resemble the complex properties of brain tissue. There are some potential candidates that, combined with other materials, could lead to this goal. For instance, injectable hydrogels^33,34^ are being studied as treatments for glioblastoma post resection. At the same time, these injectable materials offer the possibility of delivering drugs in a sustained release manner^35,36^. Even if their viscoelastic properties have not yet been compared directly to those of the brain, the injectability of these materials is promising in order to replace currently stiff and hard materials like Gliadel wafers^37^, currently used clinically to fill postsurgery cavities in the brain tissue. In brain organoid applications, the most widely used material for scaffolds is Matrigel. The limitations of this material are well-known^38^: batch-to-batch fluctuations and its constituents would make its FDA approval difficult for clinical applications, and it is a high-cost material. Therefore, there is a necessity to find new materials to substitute Matrigel. For other applications, such us brain–machine interfaces, poly(3,4-ethylenedioxythiophene) with polystyrene sulfonate (PEDOT:PSS) is the most widely used conducting polymer. At the same time, PEDOT:PSS is stable over months, and reduces the impedance by increasing the effective area for ionic–electronic transduction, making this biomaterial a so far unbeatable to improve recording and stimulation when used as a coating in neural probes for brain–machine interfaces^39^. PEDOT:PSS helps in decreasing the exposed stiffness to the brain when used as coating materials for neural probes. However, PEDOT:PSS is still orders of magnitude stiffer than the cerebral matter. This stiffness mismatch is accelerating the search for softer functional materials.

## Prospective and future directions

In conclusion, several critical shortfalls must be addressed to enable the creation of useful and reliable brain-tissue-like biomaterials. The viscoelastic moduli, ultimate toughness, tensile strength, poroviscoelastic responses, energy dissipation, adhesion forces, and solute diffusivity of the brain need to be mapped at all scales, from the bulk to the nanoscale, under physiological conditions. Such studies would enable the development of next-generation biomaterials resembling a wide-ranging set of physical properties of the brain. In this regard, it is essential that the brain biomechanics community strengthen collaborations to perform round robin tests and design standardized protocols. In addition, degradation studies of implants need to be designed to mimic the particular physiological conditions of the brain. We believe that these challenges are, while certainly difficult to tackle, addressable with technologies available today through cohesive interdisciplinary efforts by materials scientists, mechanical engineers, biologists, and clinician scientists. Looking forward, we hope to inspire and help researchers to fabricate new biomaterials that can recapitulate the mechanical, physical, and diffusion properties of the brain.
